# Short Peptides Protect Fibroblast-Derived Induced Neurons from Age-Related Changes

**DOI:** 10.3390/ijms252111363

**Published:** 2024-10-22

**Authors:** Nina Kraskovskaya, Natalia Linkova, Elena Sakhenberg, Daria Krieger, Victoria Polyakova, Dmitrii Medvedev, Alexander Krasichkov, Mikhail Khotin, Galina Ryzhak

**Affiliations:** 1 Institute of Cytology RAS, Tikhoretski Pr., 4, St. Petersburg 194064, Russia; ninakraskovskaya@gmail.com (N.K.); lenasakhenberg@yandex.ru (E.S.); daryamalikova@gmail.com (D.K.); h_mg@mail.ru (M.K.); 2 St. Petersburg Research Institute of Phthisiopulmonology, Ligovskii Pr., 2−4, St. Petersburg 191036, Russia; vopol@yandex.ru; 3 St. Petersburg Institute of Bioregulation and Gerontology, 3 Dynamo Ave., St. Petersburg 197110, Russia; rsc-ide@yandex.ru (D.M.); galina@gerontology.ru (G.R.); 4 The Department of Social Rehabilitation and Occupational Therapy, St. Petersburg Medical and Social Institute, Kondratievsky St., 72A, St. Petersburg 195271, Russia; 5 Department of Radio Engineering Systems, Saint Petersburg Electrotechnical University ‘LETI’, 5F Prof. Popova St., St. Petersburg 197376, Russia; krass33@mail.ru

**Keywords:** tripeptides, induced neurons, aging, oxidative DNA damage, dendrites, morphology, mitochondria, lysosomes, short peptides

## Abstract

Neurons become more vulnerable to stress factors with age, which leads to increased oxidative DNA damage, decreased activity of mitochondria and lysosomes, increased levels of p16, decreased LaminB1 proteins, and the depletion of the dendritic tree. These changes are exacerbated in vulnerable neuronal populations during the development of neurodegenerative diseases. Glu-Asp-Arg (EDR) and Lys-Glu-Asp (KED), and Ala-Glu-Asp-Gly (AEDG) peptides have previously demonstrated neuroprotective effects in various models of Alzheimer’s disease. In this study, we investigated the influence of EDR, KED, and AEDG peptides on the aging of fibroblast-derived induced neurons. We used a new in vitro cellular model of human neuronal aging based on the transdifferentiation of aged dermal fibroblasts from elderly donors into induced cortical neurons. All peptides promote the arborization of the dendritic tree, increasing both the number of primary processes and the total length of dendrites. Tripeptides have no effect on the activity of mitochondria and lysosomes and the level of p16 protein in induced neurons. EDR peptide reduces oxidative DNA damage in induced neurons derived from elderly donor fibroblasts. Short peptides partially protect induced neurons from age-related changes and stimulate dendritogenesis in neurons. They can be recommended for use as neuroprotective agents.

## 1. Introduction

Neurons, like other cells in our body, are subject to age-related changes. These changes occur during normal aging and are exacerbated in vulnerable neuronal populations in neurodegenerative diseases like Alzheimer’s disease (AD) [1]. Most neurodegenerative diseases, in spite of the different etiologies, are characterized by the progressive death of neurons in certain areas of the brain. Cellular stress of various origins contributes to degenerative changes in neurons, such as oxidative DNA damage [2], mitochondrial dysfunction [3], lysosomal dysfunction [4], and calcium dysregulation [5]. Despite the huge amount of research conducted to study the mechanisms of normal brain aging and neurodegenerative diseases, effective approaches and drugs to prevent or cure these diseases have not yet been developed [6].

To protect neurons from such damage during aging, and in the complex therapy of neurodegenerative diseases, neuroprotective agents of various natures are used. These can be small molecules and antibodies for disease-modifying treatment [7,8]; plant-based neuroprotective agents [9,10] and natural product agents that promote the trophic state and plasticity of neurons, inhibit the accumulation of reactive oxygen species, and block excitotoxicity that are also considered in AD treatment strategies [11].

In the context of such a strategy, peptide bioregulators also can act as potential neuroprotective remedies [12]. According to their composition, they can be divided into the following two groups: polypeptide complexes isolated from the brain of cattle or pigs (cortexin, Cerebrolysin, etc.) and short synthetic peptides (semax, cortagen, pinealon, vesugen, etc.). Short synthetic peptides like KED, EDR, and AEDG have a neuroprotective effect similar to polypeptide complexes, but in lower concentrations [13]. The structures and characteristics of these peptides are presented in Table 1. KED, EDR, and AEDG peptides were isolated from the polypeptide complexes of the vessels, cerebral cortexes, and pineal glands of calves, respectively [14]. These short peptides are the active beginnings of polypeptide complexes.

Our previous studies have demonstrated EDR and KED’s neuroprotective properties in cellular and mouse models of Alzheimer’s disease [15,16]. In addition, there is evidence of the effect of these peptides on apoptosis in neurons under conditions of cellular stress. The stimulating effect of EDR and AEDG peptides on the short-term and long-term memory of bees also has been previously demonstrated [17]. In a study of the effect of peptides on the neuronal differentiation of human periodontal ligament stem cells (hPDLSCs), it was shown that the AEDG peptide positively affects neurogenic differentiation gene expression and protein synthesis in human gingival mesenchymal stem cells [18]. Moreover, peptides AEDG and KED prevented cellular senescence in hPDLSCs during long-term cell cultivation detected by p16 and p21 markers [19].

To evaluate the effect of peptide bioregulators, we used a new model of neuronal aging based on direct reprogramming of aged fibroblasts into induced neurons [20,21,22]. This approach allows to preserve age-related changes occurring in aged cells. Since previous studies have demonstrated a positive therapeutic effect of peptide bioregulators on cellular and animal models of neurodegenerative diseases, a logical continuation of these studies is to test these compounds on the aging model of induced human neurons. In particular, assessing the effect of these compounds on processes associated with aging and neurodegenerative changes in cells, such as the activity of lysosomes and mitochondria, marker proteins of cellular aging, such as p16, laminB1 and 8-OHDG—a marker of the oxidative DNA damage and morphology of induced neurons after application of short peptides for 10 days.

The aim of this study is to evaluate the effect of EDR, KED, and AEDG peptides on markers of age-related changes in a new model of neuronal aging based on the direct conversion of fibroblasts from elderly donors into induced neurons.

## 2. Results

**Figure 1:**
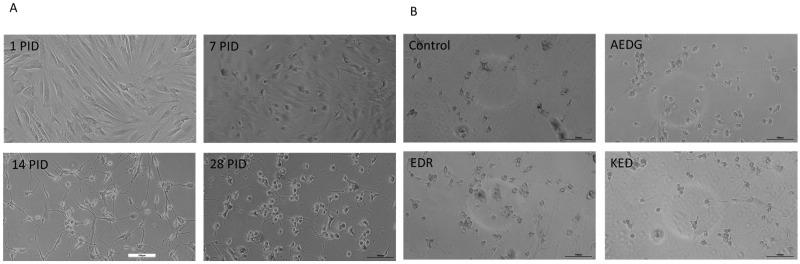
Microphotographs of dermal fibroblasts in the process of transdifferentiation in the neuronal direction (**A**) and after the application of short peptides (**B**). (**A**) Images illustrating changes in the morphology of induced neurons derived from dermal fibroblast lines of elderly donors at 0 days in vitro (DIV), 7 DIV, 14 DIV, and 28 DIV. Light microscopy, ×10. Scale bars correspond to 100 µm. (**B**) Images illustrating morphology of induced excitatory neurons at 38 DIV after application of short peptides AEDG, EDR, and KED. Light microscopy, ×10. Scale bars correspond to 100 µm. PID—post-infection day. EDR—Glu-Asp-Arg, KED—Lys-Glu-Asp, AEDG—Ala-Glu-Asp-Gl.

During the process of differentiation in the neuronal fate, dermal fibroblasts obtained from the skin of elderly donors gradually lose their characteristic elongated spindle-shaped form and begin to round out and form processes 7 days after transduction with lentiviruses (post-infection day—PID). By day 14, the cell morphology becomes more complex, and by day 28, cells are characterized by long processes and form a developed network of contacts between cells (Figure 1A). The application of short peptides in 10 ug/mL concentration for 10 days does not negatively affect the morphology of induced neurons (Figure 1B).

**Figure 2:**
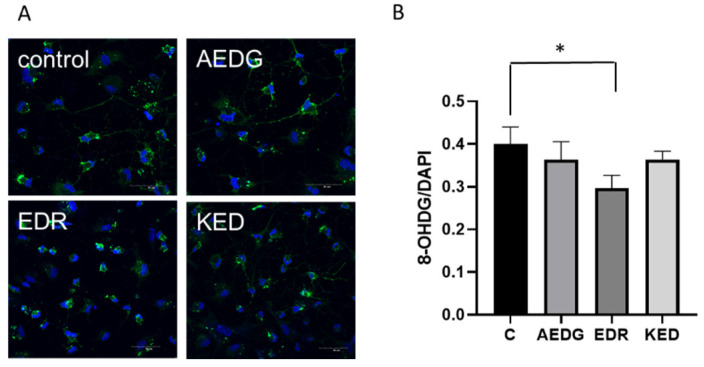
Microphotographs illustrating the analysis of the 8-OHdG level in induced neurons after short peptide application. (**A**) Immunofluorescent staining with antibodies to 8-OHdG (secondary antibodies Alexa 488, green) and nuclei visualization with DAPI (blue) dye in the control cells and treated with short peptides AEDG, EDR, and KED in induced neurons derived from dermal fibroblast lines of elderly donors. Confocal microscopy, ×40. Scale bar 50 μm. (**B**) Histogram illustrating the 8-OHdG level calculation in induced neurons derived from dermal fibroblasts of elderly donors. Data are presented as mean ± SEM. * *p* < 0.05—in comparison with the control group (C).

DNA damage is a well-known mediator of cellular senescence in mitotic cells, mainly due to replication errors and the influence of factors such as oncogenic and oxidative stress [23]. Among more than 30 different base modifications and other types of oxidative DNA damage, the most common is 8-hydroxydeoxyguanosine (8-OHdG), which leads to mutations through the formation of GC-TA transversions [24]. To evaluate if short peptides protect induced neurons from oxidative DNA damage, we measured the 8-OHdG level. The relative value of the fluorescent staining intensity of 8-OHdG divided by the signal from DAPI in the control group of induced neurons obtained from the fibroblasts of elderly donors in the absence of peptides was 0.40 ± 0.04 relative units. After the addition of the AEDG peptide, the level slightly decreased by 10% to 0.36 ± 0.04; after the addition of the EDR peptide, it statistically significantly decreased by 23% to 0.31 ± 0.03; and after the addition of the KED peptide, it decreased by 15% to 0.34 ± 0.03. Based on the obtained results, it can be concluded that the effect of the EDR peptide with a value very close to significant (*p* = 0.0566) contributes to the reduction of oxidative damage to DNA in cells during aging (Figure 2A,B).

**Figure 3:**
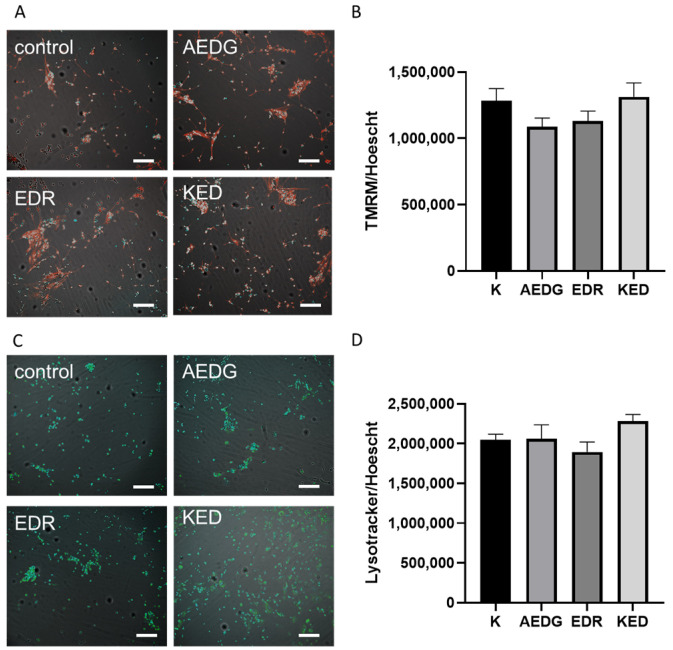
Microphotographs illustrating the analysis of TMRM and lysotracker levels in induced neurons after the application of short peptides. (**A**) Live imaging staining with TMRM dye (red) and nuclei visualization with Hoechst dye (blue). Scanning confocal microscopy, ×10. Scale bar 200 um. (**B**) Histogram illustrating the mitochondrial activity detected by the TMRM in induced neurons derived from dermal fibroblast lines of elderly donors. Data are presented as mean ± SEM. (**C**) Live imaging staining with lysotracker dye (green) and nuclei visualization with Hoechst dye (blue). Scanning confocal microscopy, ×10. Scale bar 200 um (**D**) Histogram illustrating the mitochondrial activity detected by the lysotracker in induced neurons derived from dermal fibroblast lines of elderly donors. Data are presented as mean ± SEM.

Neuronal cells require a large amount of energy; thus, mitochondrial malfunction is considered a serious factor in neuronal dysfunction. Cellular aging also involves not only mitochondrial impairment but also a marked deterioration in proteostasis, which is the cellular network that governs protein life from synthesis to degradation [25]. In neurodegenerative diseases, which typically involve widespread neuronal cell death, affected neurons also show an increase in dysfunctional lysosomes [26]. Thus, we access the functional activity of mitochondria and lysosomes in induced neurons obtained from aged donors. The ratio of the relative fluorescence intensity of the TMRM signal divided by the number of cell nuclei detected by the fluorescent dye in induced neurons was 1,282,919 ± 92,318 relative units in the control. After the application of the AEDG peptide, it slightly decreased by 16% to 1,086,801 ± 65,835 relative units; after the application of the EDR peptide, it slightly decreased by 12% to 1,130,588 ± 75,002 relative units; and after the application of the KED peptide, it remained virtually unchanged and was 1,313,458 ± 104,593 (Figure 3A,B). A similar analysis was performed to investigate the lysosomal activity in induced neurons. In the absence of peptides, the lysotracker fluorescence intensity value was 2,046,932 ± 71,737. After the application of the AEDG peptide, the fluorescence intensity remained unchanged and was 2,060,850 ± 175,204; after the application of the EDR peptide, it decreased by 8% to 1,895,572 ± 123,632; and after the application of the KED peptide, it decreased slightly by 11% to 2,282,061 ± 82,881 (Figure 3C,D). Thus, as in the case of mitochondria, statistically significant differences in the effect of peptides on lysosome activity were not revealed.

**Figure 4:**
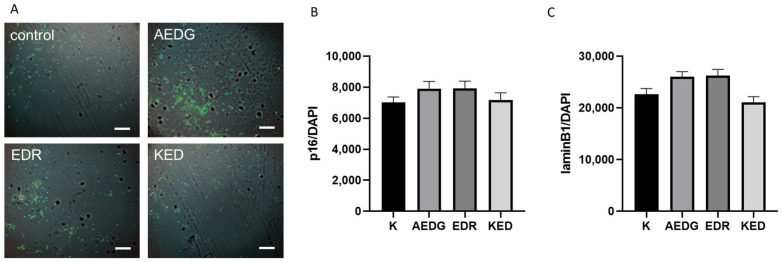
Microphotographs illustrating the analysis of p16 and laminB1 levels in induced neurons after application of short peptides. (**A**) Immunofluorescent staining of induced neurons with antibodies to laminB1 (secondary antibodies Alexa 488, green), p16 (secondary antibodies Alexa 555, red), and nuclei visualization with DAPI (blue) dye in the control cells and cells treated with short peptides AEDG, EDR, and KED. Scanning confocal microscopy, ×10. Scale bar 200 um. Histogram illustrating the level of p16 protein (**B**) and lamin B1 protein (**C**) expression in induced neurons derived from dermal fibroblast lines of elderly donors. Data are presented as mean ± SEM.

The p16 protein, also known as INK4a, is a cyclin-dependent kinase (CDK) inhibitor that plays an important role in cell cycle regulation. Although neurons are non-dividing cells in the G_0_ phase, p16 is detected in some neurons and may play a role in the aging of this cell type. However, the mechanisms by which p16 influences neuronal aging are still not fully understood. However, p16 expression has been detected not only in the brains of patients with amyotrophic lateral sclerosis/motor neuron disease (ALS/MND) but also in control samples [27]. This suggests that p16 may be activated in neurons in response to various stresses, including natural aging, and not just neurodegenerative changes [28]. Similar work showed the expression of this protein in neurons both in healthy elderly people in 5% of cases and in patients with Alzheimer’s disease, with the latter having an increase in the number of positively stained neurons to 20% [29]. The loss of lamin B1 is an aging-related biomarker, but this is not easily detectable in the aged brain using immunostaining because mature neurons express low levels of lamin B1 [30]. Senescent cells undergo nuclear morphological changes, such as shape and size changes and decreased lamin B1 expression [31]. Long-term cultured neurons from mice and rats exhibit abnormal nuclear morphologies and decreased lamin B1 levels, indicating that nuclear morphological changes occur in aged neurons [32]. Interestingly, the balance is also important in aging because lamin B1 has been linked to age-related neurodegenerative diseases. Therefore, we also analyzed the effect of short peptides on the expression level of laminB1 and p16 in our model. In induced neurons obtained from the fibroblasts of elderly donors, the level of lamin B1 expression in the control was 22,650 ± 1091. After the application of the AEDG peptide, it slightly increased by 14% to 26,031 ± 991.4, after the application of the EDR peptide, it slightly increased by 16% to 20,726 ± 1235; and after the application of the KED peptide, it slightly decreased by 7% to 21,082 ± 1081. Thus, no statistically significant differences were revealed in the effect of peptide bioregulators on the level of laminB1 protein expression (Figure 4A,C). The level of p16 expression in the control was 7021 ± 352.4. After the application of the AEDG peptide, the protein level slightly increased by 12% to 7889 ± 478.0; after the application of the EDR peptide, it also slightly increased by 12% to 7927 ± 464.5; and after the application of the KED peptide, it did not change and was 7179 ± 458.1. Thus, no statistically significant differences were found in the effect of peptide bioregulators on the level of p16 protein expression either (Figure 4).

**Figure 5:**
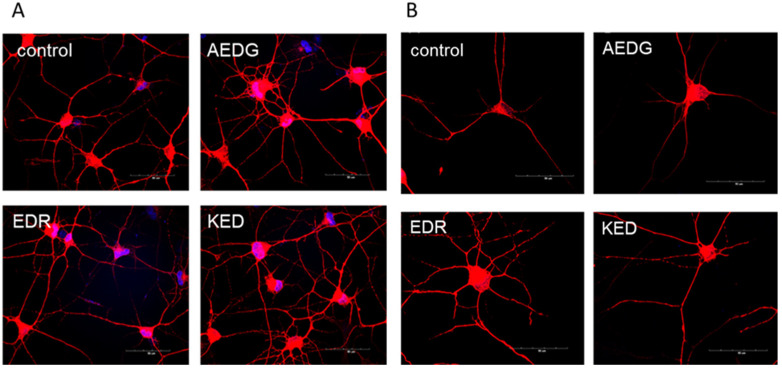
Microphotographs illustrating the population of induced neurons (**A**) and morphology of individual neurons (**B**) after application of short peptide application. Immunofluorescent staining for the neuronal marker protein MAP2 (secondary antibodies Alexa 555, red) and nuclei visualization with DAPI (blue) dye of induced neurons derived from dermal fibroblast lines of elderly donors. Confocal microscopy, ×60 (**A**). Scale bar 50 μm.

Normal aging does not involve a massive loss of nerve cells. However, structural changes occur in neurons, including a decrease in the number and length of dendrites; many dendritic processes are lost, and the number of axons decreases. It is suggested that these changes are likely to contribute significantly to the behavioral impairments and cognitive decline that often accompany normal aging [33]. We also noted that in the presence of short peptides, the induced neurons visually form a more developed network of processes (Figure 5), so we also analyzed the morphology of the dendritic tree in a control group and after the application of short peptides in NeuronStudio software V6.

**Figure 6:**
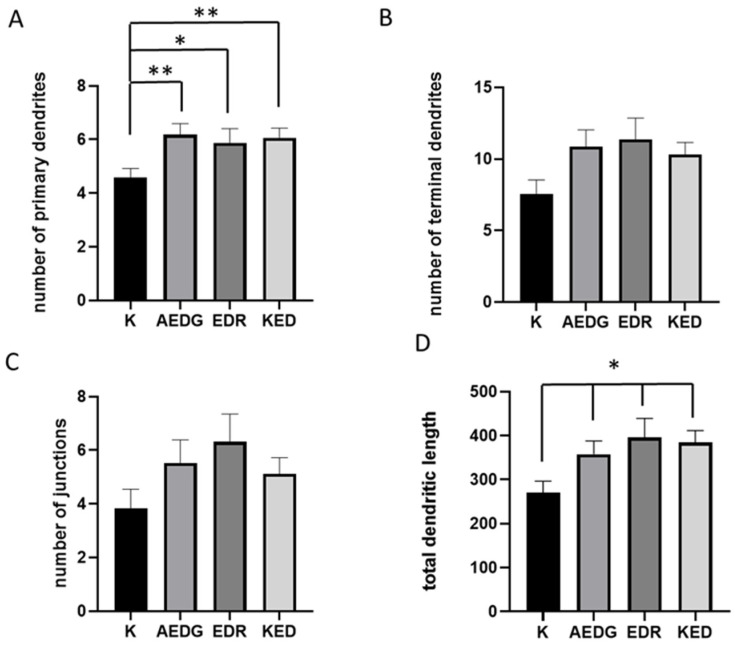
Histogram illustrating the number of primary processes (**A**), the number of branching points (**B**), the number of terminal processes (**C**), and the total length of dendrites (**D**) in the control and after application of peptide bioregulators in induced neurons obtained from dermal fibroblast lines and elderly donors. Data are presented as the mean ± SEM * *p* < 0.05. ** *p* < 0.01.

The obtained analysis demonstrated (Figure 6) that the number of primary processes in induced neurons in the control was 4.59 ± 0.3. After the application of the AEDG peptide, a statistically significant increase in the number of processes by 34% to 6.18 ± 0.4 (*p* = 0.0046) is observed; after the application of the EDR peptide, a statistically significant increase in the number of processes to 5.87 ± 0.5 (*p* = 0.0208) is also observed, which is 28%; and after the application of the KED peptide, a statistically significant increase by 32% to 6.06 ± 0.3 (*p* = 0.0093) is observed. Next, the number of branching points was analyzed. In the control group, it was 3.82 ± 0.7. After the application of the AEDG peptide, a slight increase in the number of branching points by 44% to 5.53 ± 0.8 is observed; after the application of the EDR peptide, a slight increase in branching points to 6.31 ± 1.0 is also observed, which is 65%; and after the application of the KED peptide, an insignificant increase of 34% to 5.12 ± 0.6 is observed. Then, the number of terminal processes was analyzed; in the control, the value of this parameter was 7.53 ± 1.0. After the application of the AEDG peptide, a statistically insignificant increase in the number of processes by 44% to 10.88 ± 1.2 is observed; after the application of the EDR peptide, a statistically insignificant increase in the number of processes to 11.38 ± 1.2 is also observed, which is 51%; and after the application of the KED peptide, a statistically insignificant increase of 37% to 10.31 ± 0.8 is observed. Finally, the total length of all processes was analyzed. In the control, its value was 270.2 ± 25.5. After the application of the AEDG peptide, a statistically significant increase in the number of processes by 32% to 357.8 ± 30 (*p* = 0.05) is observed; after the application of the EDR peptide, a statistically significant increase in the number of processes to 395.9 ± 43 is also observed, which is 46% (*p* = 0.0332); and after the application of the KED peptide, a statistically significant increase of 42% to 384 ± 27 (*p* = 0.0148) is observed. Thus, it can be concluded that all the studied peptide bioregulators, AEDG, EDR, and KED, are able to positively influence the morphological structure of the dendritic tree of induced neurons due to the formation of primary dendrites, increasing their number by 34%, 28%, and 32%, respectively, and also contribute to an increase in the total length of dendrites by 32%, 46%, and 42%, respectively.

## 3. Discussion

Scientists attribute the lack of effective methods for treating neurodegenerative diseases with clinically relevant models for studying neuropathology in humans [34]. The creation of effective therapy is possible only with an understanding of the molecular–cellular interactions that lead to pathological changes in aged neurons. In this regard, there is an urgent need to develop methodological and technological approaches for modeling the studied degenerative changes in human neuronal cells, taking into account age and individual characteristics [35]. For most sporadic forms of neurodegenerative diseases, the main risk factor is advanced age [36]. Therefore, studying the effectiveness of potential drugs and neuroprotectors in preclinical studies on models that adopt age-related characteristics of human neurons is extremely important. At the same time, the biological aging of human neurons is an extremely complex process for modeling. Great hopes were placed on rapid progress in understanding the causes of neurodegenerative changes in neurons with the advent of induced pluripotent stem cell (iPSC) technology. However, it quickly became clear that the populations of induced neurons obtained through iPSCs are rejuvenated during the transition through the pluripotent state [37,38], and it is necessary to additionally “age” the cells to identify the pathological phenotype [39,40,41]. The ability to directly convert fibroblasts from elderly donors into induced neurons represents a unique opportunity to study age-related diseases, since iNs retain a wide range of aging features that are not present in their iPSC-derived counterparts.

It has been previously demonstrated that in induced neurons from elderly donors, compared to young donors, there is increased mitochondrial fragmentation and a decrease in their length, as well as a decrease in the mitochondrial membrane potential [42]. Also, in this model, higher levels of ROS and higher oxidative stress were shown [43], as well as impaired nucleocytoplasmic compartmentalization [22]. In addition, the literature data convincingly demonstrate the application of this technique for modeling age-associated changes in the development of neurodegenerative diseases like Alzheimer’s disease [29,44,45,46] and Huntington’s disease [34,47,48].

In the present study, the effectiveness of neuro- and geroprotective drugs—peptide bioregulators that have proven successful in preclinical studies of cellular and animal models of AD—was assessed using a new cellular model of neuronal aging. Using a direct reprogramming protocol based on the use of micro-RNA and transcription factors that govern the differentiation of skin fibroblasts into induced neuronal cells, induced neurons were obtained from the fibroblasts of elderly donors, and an analysis of age-associated changes in these cells was carried out, including oxidative DNA damage, changes in the activity of mitochondria and lysosomes, and age-related markers of aging—proteins p16 and laminB1. However, no effects of peptide application on mitochondrial and lysosome activity were detected. This is probably due to significant individual differences in cellular responses among donors. Finally, all three peptide bioregulators had a positive effect on the morphology of the dendritic tree, increasing both the number of primary processes and the total length of dendrites in induced neurons obtained from fibroblasts of elderly donors, which may indicate their ability to influence dendritogenesis. For EDR and AEDG peptides, the mechanism of the neuroepigenetic regulation of gene expression can be implemented through interaction with specific sites of double-stranded DNA in promoters of genes associated with AD pathogenesis. KED peptide can regulate gene expression by interacting with histone proteins and/or the nucleosome [12]. Thus, these peptides can regulate the expression of genes, which is connected with dendrite growth, but cannot regulate mitochondrial or lysosomal genes. The effects we observed are comparable to the results obtained in the study of other peptide regulators, such as Cerebrolysin and synthetic analog of ACTH 4-10 (Semax). It is known that these peptides have a nootropic effect and protect neurons from damage under conditions of cellular stress [49,50]. Cerebrolysin has also been shown to protect neurons from d-galactose-induced oxidative stress [51].

In particular, Cerebrolysin also stimulates the process of the neurite growth of embryonic chicken telencephalon [52]. Semax at concentrations of 10 and 100 µM stimulates the activity of glutamatergic synapses in neural networks, as well as the ability of the peptide to effectively modulate the short-term plasticity in sensory synapses in dorsal root ganglion (DRG) and dorsal horn (DH) neurons [53]. It was also demonstrated that Semax (100 μM) and its Pro-Gly-Pro fragment (20 and 100 μM) delayed the development of calcium dysregulation and reduction of the mitochondrial potential in cultured cerebellar granule cells under conditions of glutamate neurotoxicity [49].

Thus, the available literature data on the effect of peptide regulators Semax and Cerebrolysin to stimulate the formation of processes are in good agreement with our data. In addition, our results on the effects of the EDR peptide on oxidative DNA damage are also consistent with previously published data on the ability of peptide bioregulators to protect neuronal cells from this pathological process. There is evidence in the literature that some peptide bioregulators can have a positive effect on the membrane potential of mitochondria, but unlike our study, this effect in Semax and its fragment is observed at significantly higher doses. Unfortunately, we did not find previously published results on the effect of peptides on the expression level of proteins p16 and laminB1 in the available literature.

Both Semax and Cerebrolysin have been widely studied as nootropic remedies that protect neurons from cellular stress of various origins. In the future, it seems interesting to evaluate the effect of KED, EDR, and AEDG peptides on the survival of induced neurons obtained from fibroblasts of elderly donors under conditions of cellular stress.

## 4. Materials and Methods

Object of this study. The project was carried out on 3 human dermal fibroblast cell lines obtained from elderly women aged 61, 66, and 68 (lines DF262, DF234, and DF4, respectively). The DF262 and DF234 lines were obtained from the cell culture collection of the Koltsov Institute of Developmental Biology of the Russian Academy of Sciences. The DF4 fibroblasts were isolated from a skin biopsy of a 68-year-old woman. The biopsy was obtained in a hospital setting. Before this, the donor underwent the necessary medical examination and signed a voluntary informed consent.

### 4.1. Cultivation of HEK293T Cells and Dermal Fibroblast Lines

HEK293T cells were used to assemble lentiviruses. Human dermal fibroblasts were used for targeted differentiation into neurons. HEK293T cells and dermal fibroblast lines were cultured in DMEM with high glucose content (Gibco, Waltham, MA, USA), with the addition of 10% fetal calf serum (HyClone, Logan, UT, USA) at a temperature of 37 °C and 5% CO_2_. In the experiments, cells from passages 3 to 9 were used with a confluence state of 70–80% for HEK239T cells and 90–95% for dermal fibroblasts.

### 4.2. Lentiviruses Production

Lentiviruses were used for efficient fibroblast transduction and a high expression of transcription factor and micro-RNA genes. They were assembled in HEK293T cells through co-transfection of the psPAX packaging vector (Addgene, #12260, Watertown, MA, USA), pMD2.G delivery vector (Addgene, #12259), and the plasmid of interest (micro-RNA, transcription factors) using the commercial chemical agent polyethyleneimine (Polysciense, #23966, Niles, IL, USA). Cells were transfected in a serum-free medium for 16 h. Then the medium was replaced with a fresh one, and HEK293T cells produced viruses for 16 h. A total of 72 h after co-transfection, the conditioned medium was collected, passed through a filter with a pore diameter of 0.45 mm, and centrifuged at 70,000× *g* for 2 h at +4 °C. The pellet was then dissolved in sterile cold phosphate-buffered saline containing 20% sucrose and aliquoted for further use.

### 4.3. Direct Reprogramming Technique

For direct reprogramming of dermal fibroblasts into excitatory neurons, we adopted a protocol developed by the group of A. Yoo for excitatory neurons [54] with some modifications [55]. Briefly, fibroblasts were cultured on a 6-well plate to ~100% confluency. Upon reaching a monolayer, the cells were transduced with 4 lentiviruses encoding the reverse transactivator of tetracycline, micro-RNA under a doxycycline-inducible promoter, and 2 transcription factors, MYT1L and NeuroD2, in the presence of hexadimethrine bromide at a final concentration of 8 μg/mL. The next day, the medium was replaced with a new one containing 1 uM rapamycin (Selleckchem, Houston, TX, USA) for cell synchronization [55]. A total of 48 h later, cells were treated with doxycycline (DOX) at a final concentration of 1 μg/mL. Then the fibroblasts were incubated for 2 days, and on the third day after the induction of micro-RNA expression, DOX was added at a concentration of 1 μg/mL and puromycin was added at a concentration of 3 μg/mL. Then the fibroblasts were incubated for 2 days in the presence of antibiotics, and on the 3rd day after the DOX and puromycin administration, the cells were transplanted onto a 96-well plate, the wells of which were pre-coated with Matrigel (Matrigel, Corning, New York, NY, USA). After 2 days of reseeding, the condition medium for fibroblasts was changed to a reprogramming medium, to which DOX and puromycin were also added at the concentrations indicated above for the 2 rounds of selection. The reprogramming medium contains Neurobasal-A supplemented with 2% B-27, 0.125 mM GlutaMAX supplement (all from Gibco, USA), 1 mM valproic acid (Sigma Aldrich, St. Louis, MO, USA), 1 μM retinoic acid (Sigma Aldrich, USA), 200 μM dibutyl cAMP (Sigma Aldrich, USA), 1 μM ISX9 (SelleckChem, Houston, TX, USA), 20 ng/mL BDNF (PeproTech, Cranbury, NJ, USA), and 20 ng/mL neurotrophin type 3 (PeproTech, USA). During the first 2 weeks of reprogramming, 1 μM DAPT (SelleckChem, USA) was added to the cells to enhance differentiation. The cells were reprogrammed for 30 days. Every 2 days, DOX was added to the cells at a concentration of 1 μg/mL, and every 4 days, it was added at a concentration of 2 μg/mL. Every week, 400 μL of fresh reprogramming medium was added to each well. At the end of differentiation, EDR, KED, or AEDR peptides were added to the induced neurons every day for 10 days at concentrations of 10 μg/mL. This concentration was chosen as the most effective according to the results of the premilitary experiment. The description of the premilitary experiment is in the Supplementary Materials (Figures S1–S3). On the 11th day after the addition of the peptides, a functional assessment of the activity of the mitochondria and lysosomes and immunofluorescence staining for neuronal markers were conducted. Cells to which peptides were not added served as a control.

### 4.4. Immunofluorescence Staining

Before immunofluorescence staining, the cells were washed from the conditioned medium with phosphate-buffered saline (PBS) and fixed in 10% formalin for 20 min at room temperature. This was followed by a series of washes with PBS and the permeabilization of cell membranes with a 0.25% Triton X-100 solution (Helikon, Moscow, Russia) in PBS for 5 min at room temperature. Then the cells were washed with PBS 3 times and incubated in a blocking solution containing 5% bovine serum albumin (Sigma-Aldrich, USA) and diluted in PBS for 1 h at room temperature to prevent the nonspecific binding of antibodies. Then the cells were incubated with primary antibodies indicated in Table 2 and diluted in a blocking solution overnight at +4 °C. Next, a series of washes with PBS solution were performed, after which the cells were incubated with secondary antibodies for 1 h at room temperature and diluted in a blocking solution. Next, a series of washes with PBS solution was performed, as well as staining with DAPI, after which the cells were fixed on a glass slide using mounting media (Cell Signaling, Danvers, MA, USA).

A live imaging analysis of mitochondrial membrane potential in the absence and presence of peptide bioregulators was performed by staining with 50 nM tetramethylrhodamine dye solution (TMRM, Invitrogen, Carlsbad, CA, USA) and 100 nM LysoTracker Green DND-26 solution (Lysotracker, Invitrogen, USA), respectively. A total of 1 μM of Hoechst 33,342 solution (Invitrogen, USA) was added to visualize the nuclei of living cells. The fluorescent signal was detected using the CQ1 cell intravital imaging system (Yokohawa, Kanagawa, Japan) based on spinning disk microscopy. For each exposure, at least 4 fields with cells were analyzed. The total signal intensity from TMRM, Lysotracker, and 8-OHDG in the field of view was divided by the signal from Hoechst and averaged over the fields of view. The image intensity analysis was performed using Image_J software (https://imagej.net/ij/).

### 4.5. Neuronal Morphology Analysis

For the morphological study, a series of images of induced neurons in TIFF format with a size of 1024 × 1024 pixels, averaged 3–4 times, were obtained. The thickness of the series of sections was, on average, 7 μm, with a step of 1 μm. A comparative assessment of the branching of induced neurons was carried out using the NeuronStudio software V6, which allows for the reconstruction of a three-dimensional image of neurons and the construction of a neuron model based on the obtained image. After constructing the neuron model, an analysis was carried out of the number of primary processes, the number of branching points of processes, the number of terminal processes of all generations, and the total length of all processes.

### 4.6. Statistics

The normality of distribution was assessed using the Shapiro–Wilk test. The significance level (α) was taken to be 0.05. The results were considered reliable if the probability (*p*) of violating the hypothesis of no differences between the samples was less than the significance level α. In this case, the significance was *p* ≤ 0.05. For analysis among several samples, one-way multivariate ANOVA was used, followed by Tukey’s post hoc test to compare means in the case of normal distribution. For analysis among several samples with deviation from normal distribution, Dunn’s test was used. The results are presented as the mean ± error of the mean for normal distribution and the median ± interquartile range in the case of deviation from normal distribution.

## 5. Conclusions

Our previous studies have revealed the neuroprotective effects of KED and EDR peptides on the Alzheimer’s disease model in vitro and in vivo [15,16]. It has also been previously shown that AEDG peptides can stimulate the differentiation of hPDLSCs into neuronal cells [18]. In addition, AEDG and KED peptides protected hPDLSC cells from aging, which is detected using p16 and p21 markers [19]. In our study, we assessed the effect of peptides on markers of age-related changes in neurons, such as p16, laminB1, and 8-OHdG, which is a marker of oxidative DNA damage, and analyzed the activity of mitochondria and lysosomes in a new model of neuronal aging based on direct reprogramming of fibroblasts from elderly donors into induced neurons. We also assessed the effect of these peptides on morphological changes in induced neurons. In our model, we did not detect the effect of peptides on the activity of mitochondria and lysosomes, as well as markers associated with aging p16 lamin B1, but showed the effect of peptides on the morphology of induced neurons. It was shown for the first time in the experiment that AEDG peptides have a neuroprotective effect in vitro comparable to KED and EDR peptides. In addition, the peptide EDR almost reliably (*p* = 0.0566) contributes to a decrease in the level of oxidative DNA damage detected using 8-OHdG. Thus, the peptides KED, EDR, and AEDG can be recommended for further study in the prevention and complex therapy of age-related and neurodegenerative diseases as agents stimulating dendritogenesis.

## Figures and Tables

**Table 1:** Peptide structures and characteristics.

Peptide	2D Structure	Characteristics
AEDG peptide (Ala-Glu-Asp-Gly)	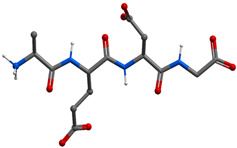	Gross formula without counterion: C_14_H_22_N_4_O_9_ Molecular weight without counterion: 390, 35 Da Counterion: trifluoroacetic acid (TFA) Polar molecule
EDR peptide (Glu-Asp-Arg)	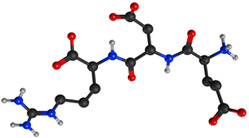	Gross formula without counterion: C_15_H_26_N_6_O_8_ Molecular weight without counterion: 418, 40 Da Counterion: acetate Polar molecule
KED peptide (Lys-Glu-Asp)	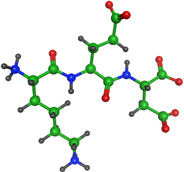	Gross formula without counterion: C_15_H_26_N_4_O_8_ Molecular weight without counterion: 390, 39 Da Counterion: acetate Polar molecule

**Table 2:** List of primary antibodies for immunofluorescent staining live imaging analysis of functional activity of mitochondria and lysosomes.

Title	Vendor	Catalog Number	Dilution
Monoclonal Anti-DNA/RNA Damage antibody	Sigma-Aldrich	SAB5200010	1/1000
P16	Cell Signaling	18769	1/800
TUJ-1	R&D Systems (Minneapolis, MN, USA)	MAB1195	1/2000
MAP2	Abcam (Cambridge, UK)	ab5392	1/1000
laminB1	Novus (Chesterfield, MO, USA)	NBP2-59783	1/100

## Data Availability

The raw and analyzed data are available upon request.

## Supplementary Materials

The supporting information can be downloaded at: https://www.mdpi.com/article/10.3390/ijms252111363/s1.
