# Supplementary material for: Short Peptides Protect Fibroblast-Derived Induced Neurons from Age-Related Changes
Source: Int J Mol Sci. 2024 Oct 22;25(21):11363. doi: 10.3390/ijms252111363 (PMC11546785; doi:10.3390/ijms252111363)
Supplement: Supplementary file 1 [file ijms-25-11363-s001.zip › ijms-3241013-supplementary.pdf]

Supplementary Materials for the article
"Short Peptides Protect Fibroblast-Derived Induced Neurons from Age-Related Changes"

The goal of this premilitary experiment was to identify the minimal effective concentration of KED, EDR and AEDG peptides for the further experiments.

Objects of the investigation: DF2 cell line - fibroblasts of the eyelid skin of a 37-year-old woman. DF4 cell line - fibroblasts of the eyelid skin of a 68-year-old woman. These cell lines were obtained from the shared research facility "Vertebrate cell culture collection" of the Institute of Cytology of Russian Academy of Science, Saint Petersburg, Russia.

Methods of investigation: Dermal fibroblast lines were cultured in DMEM with high glucose content (Gibco, USA) with the addition of 10% fetal calf serum (HyClone, USA) at a temperature of 37 °C and 5% CO2. cells from passages 3 to 9 were used with a confluence state of 90-95%. It was the positive control. The viability of positive control cells by MTT test data accepted for 100%. The negative control is the stress model, "depleted cultural medium". In this group cells cultured in the medium without the addition of FBS. The viability of cells in negative control group was near 40-53% from the positive control. Peptides were added in the depleted cultural medium in 5th various concentrations (Figures S1-S3 Supplementary Materials). MTT test was done in the 4th days of cultivation in all groups. At the end of the cultivation period, the medium was changed to a medium with MTT (5 mcg / ml), 100 mcl per well. The tablet was placed in a CO2 incubator for 3 hours, after which the medium was selected, and 100 µl of DMSO solution was added to each well and the resulting formazan was extracted for 5 minutes with constant shaking of the tablet. The optical density of the resulting formazan solution in DMSO was measured using a Varioskan LUX spectrophotometer (Thermo Scientific, USA) at a wavelength of 570 nm. One-way analysis of variance (one-way ANOVA) was used to determine the differences between groups. When differences were detected ($p < 0.05$), a posteriori analysis was then performed using the Tukey criterion. All experimental groups were compared with the negative control group. It was done 15 measurements in each group.

Results. KED peptide statistically significantly increased the viability of DF2 and DF4 cells in concentration 1 µg/ml, 10 µg/ml and 100 µg/ml. The strongest effect of KED peptide was verified in the concentration of 10 µg/ml (Figure S1A,B in Supplementary Materials).

EDR peptide statistically significantly increased the viability of DF2 cells only in concentration 10 µg/ml (Figure S2A in Supplementary Materials). EDR peptide statistically significantly increased the viability of DF4 cells in concentration 10, 100 and 1000 µg/ml (Figure S2B in Supplementary Materials). The strongest effect of EDR peptide was verified in the concentration of 10 and 100 µg/ml (Figure S2B in Supplementary Materials).

AEDG peptide statistically significantly increased the viability of DF2 cells in concentration 10, 100 and 1000 µg/ml (Figure S3A in Supplementary Materials). The effect of AEDG peptide in these 3 concentrations was equal (Figure S3B in Supplementary Materials). AEDG peptide statistically significantly increased the viability of DF4 cells in concentration 10 and 100 µg/ml (Figure S3B in Supplementary Materials). The effect of AEDG peptide in these 2 concentrations was equal (Figure S3B in Supplementary Materials).

A

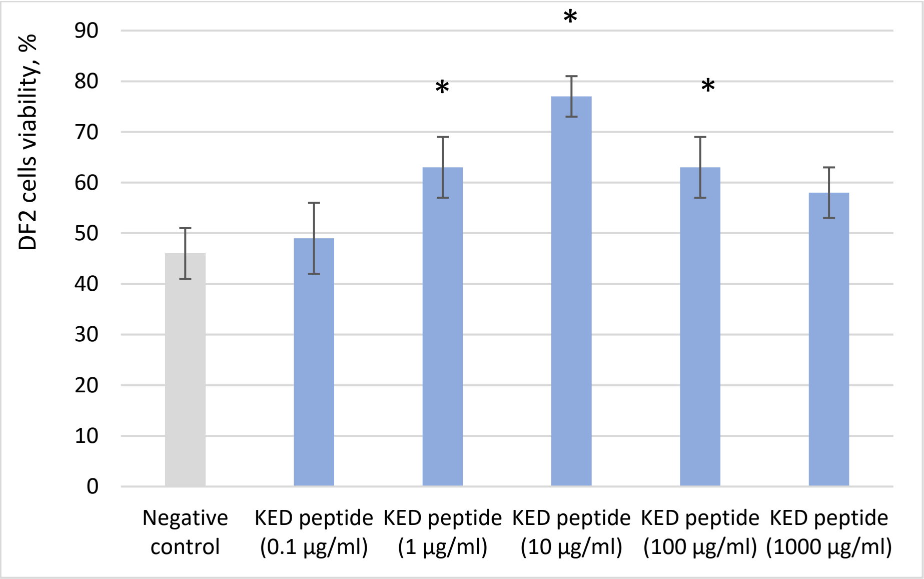

B

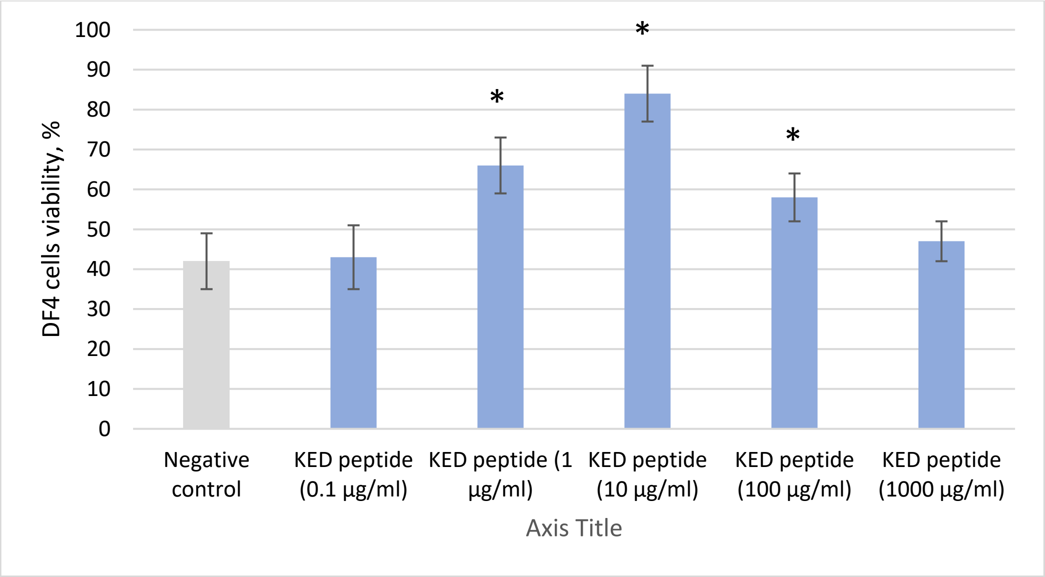

Figure S1. Supplementary materials. The influence of KED peptide in different concentrations on the viability of human dermal fibroblasts, MTT test. A – DF2 cell line, B - DF4 cell line.

* - p <0.05 in comparison with negative control (positive control is described as 100% cell viability).

A

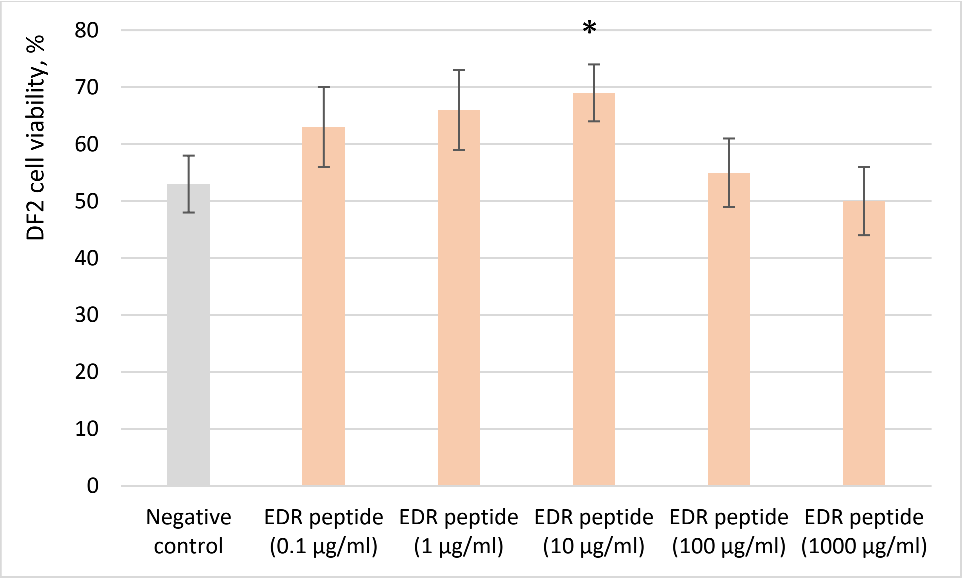

B

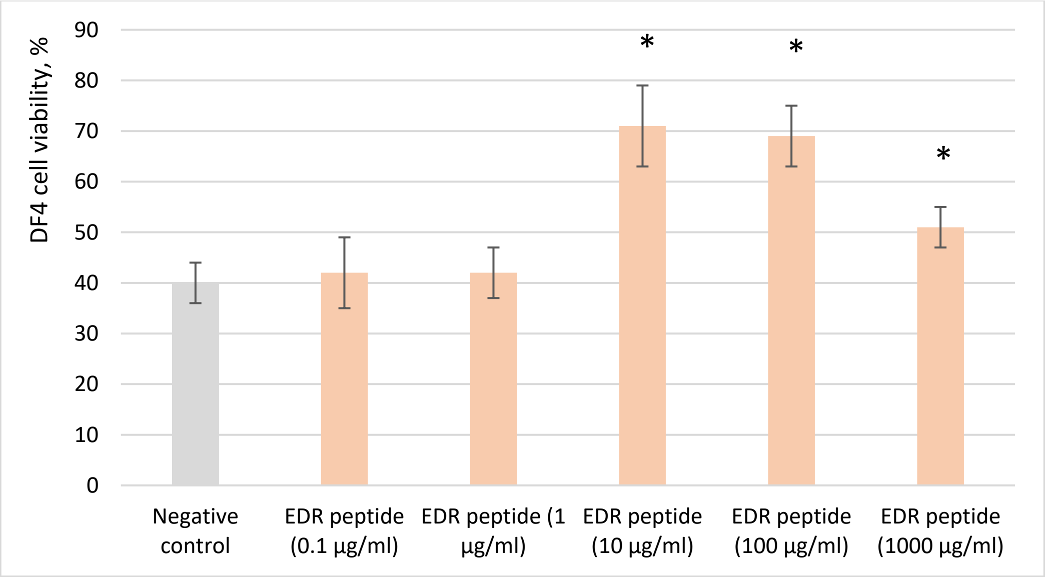

Figure S2. Supplementary materials. The influence of EDR peptide in different concentrations on the viability of human dermal fibroblasts, MTT test. A – DF2 cell line, B - DF4 cell line.

* - $p < 0.05$ in comparison with negative control (positive control is described as 100% cell viability).

A

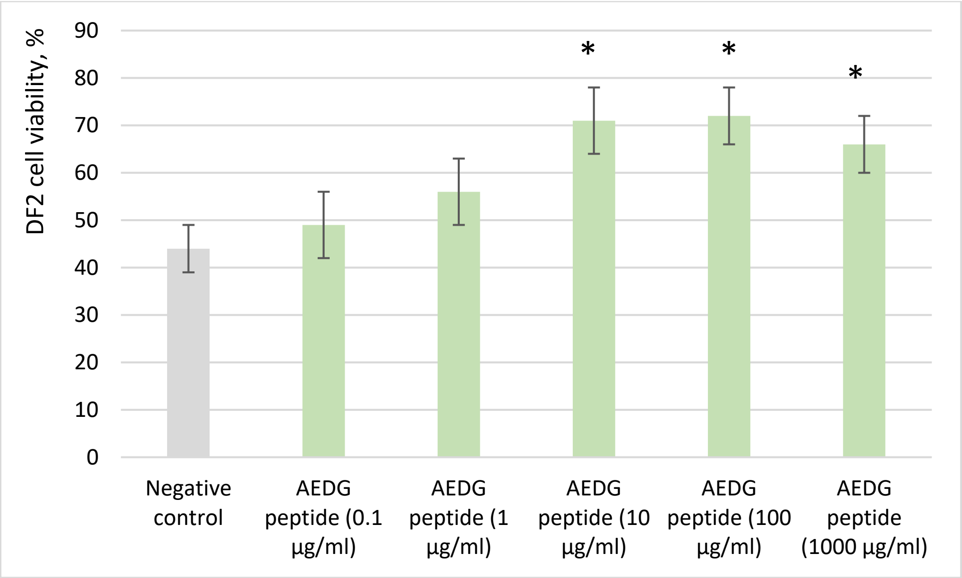

B

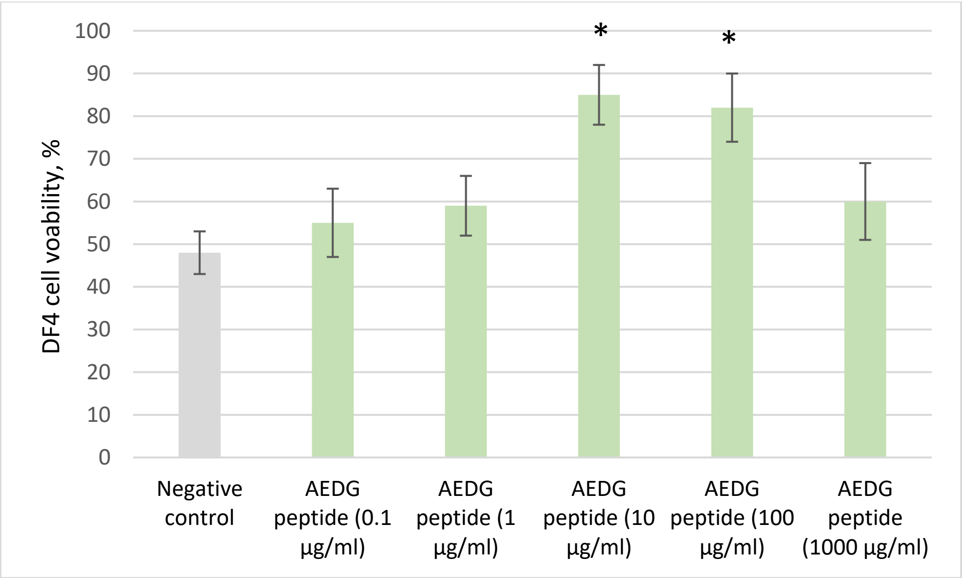

Figure S3. Supplementary materials. The influence of AEDG peptide in different concentrations on the viability of human dermal fibroblasts, MTT test. A – DF2 cell line, B - DF4 cell line.

* - p <0.05 in comparison with negative control (positive control is described as 100% cell viability).

Conclusion. The results of this premilitary experiment showed that the minimal effective concentration of KED, EDR and AEDG peptides for human skin fibroblasts is 10 µg/ml. On this reason we use this concentration in the main experiments in the article “Short peptides protect fibroblasts-derived induced neurons from age-related changes”.
